# Exercise-Induced Hypoglycemic Hemiplegia in a Child with Type 1 Diabetes: A Rare Find with Multiple Potential Causative Mechanisms

**DOI:** 10.1155/2011/529097

**Published:** 2011-11-17

**Authors:** M. Constantine Samaan, Abeer Alassaf, Jonathan DellaVedova, Trisha Murthy

**Affiliations:** ^1^Division of Pediatric Endocrinology, Department of Pediatrics, McMaster Children's Hospital, McMaster University, 1200 Main Street West, HSC-3A57, Hamilton, ON, Canada L8N 3Z5; ^2^Division of General Pediatrics, Department of Pediatrics, McMaster Children's Hospital, McMaster University, 1200 Main Street West, HSC-3A57, Hamilton, ON, Canada L8N 3Z5

## Abstract

A 10-year-old boy known to have type 1 diabetes presented to the emergency department with history of sudden onset of right-sided hemiplegia after exercise. He did not respond to oral glucose administration, but had an almost immediate resolution of symptoms with intravenous bolus of dextrose. Hemiplegic hypoglycemia is a rare complication in diabetic children, mostly affects the right side of the body, and is rarely recurrent. Children have normal brain imaging and angiography testing, and electroencephalogram may show slow-wave activity. The recovery takes place within 24 hours, and the prognosis is excellent with no focal neurological deficits noted. Our patient responded within minutes to intravenous dextrose, which is unusual and has not been reported previously. The mechanisms leading to development of hypoglycemic hemiplegia are unclear, but may involve effects of hypoglycemia on intracellular signaling pathways or molecules on motor neurons, as recent studies have shown normal brain cell glucose uptake and metabolism in hypoglycemia. While hypoglycemic hemiplegia is rare, it is a frightening experience to caregivers, and efforts should concentrate on its prevention by preventing hypoglycemia.

## 1. Introduction

Hypoglycemia results in coordinated neuroendocrine and autonomic responses, in an effort to avoid its deleterious effects. These responses include stimuli to trigger alerts to increase oral carbohydrate intake and avoid incapacitation and coma.

The presentation of hypoglycemia in diabetic children with motor manifestations is quite rare, and causative mechanisms are a matter of speculation. We describe a patient who presented with right-sided hemiplegia following a period of physical activity and recovered within minutes of intravenous glucose administration.

## 2. Case Report

A 10-year-old boy known to have type 1 diabetes presented with history of sudden onset of right-sided hemiplegia. His type 1 diabetes was diagnosed eight months prior to presentation, and he is on insulin NPH and Humalog at 0.75 units/kg/day. His most recent HbA1c was 6.7% few weeks prior to presentation reflecting excellent glycemic control; there is no history of recurrent or significant hypoglycemic events. There was no history of irritability, weakness, poor concentration, tremor, sweating, or seizures. 

He had no history of infection, syncope, chest pain, palpitations, cyanosis, abnormal movement, headache, diplopia, head injury, or drug ingestion. He has no other medical problems, and there is no family history of arrhythmias, seizures, sudden death, or diabetes. His mother has hyperthyroidism. 

On the day of presentation, he had his recommended carbohydrate intake for meals and snacks and then started swimming for four hours. Blood glucose level half way through showed hypoglycemia, with blood glucose of 2.9 mmol/L. He was treated with 15 grams of carbohydrates using juice and cookies, and he continued with his activity. 

Two hours later, he took 60 grams of carbohydrates with his meal, and his short-acting insulin was reduced by 25%. His blood glucose was checked as his mother noted he looked pale, which dropped again to 3.1 mmol/L, and 15 grams of carbohydrates was given similar to the above snack. In both events, he was unaware he was hypoglycemic.

Shortly afterwards, he developed flaccid paralysis of the right side of the body. He remained alert throughout the event and was distressed by his inability to move the right arm and leg. He received another snack of 15 grams of carbohydrates, with no improvement. 

Emergency medical services were contacted, and on the way to hospital, he received intravenous bolus of dextrose. This led to the resolution of the symptoms within minutes. 

In hospital, his physical examination was normal including GCS 15/15; he was oriented and had normal neurological exam. His pulse rate was 80/minute with sinus arrhythmia, and the rest of his exam was normal. 

His blood glucose ranged between 4.6 and 7.4 mmol/L. All his investigations including complete blood count and electrolytes were normal. His ECG showed sinus arrhythmia with no other abnormality. 

His insulin dose was reduced by 20% for the next 24 hours and was increased by 10% daily back to baseline. He did not have further hypoglycemic episodes, and neurological exam remained normal. The chronology of the events on the day, with physical activity precipitating the hypoglycemic episodes and tying treatment of hypoglycemia to complete recovery of neurological function, led to withholding of brain magnetic resonance imaging, the Holter cardiac monitoring, and electroencephalogram. 

## 3. Discussion

This 10-year-old boy presented with exercise-induced hypoglycemia with right-sided hemiplegia, which is likely due to increased carbohydrate requirements in face of increased physical activity, relative excess of insulin, and hypoglycemia unawareness. He responded poorly to oral glucose administration, but his focal neurological deficit resolved minutes after intravenous dextrose administration. There was no evidence of arrhythmias, transient ischemic attack, drug ingestion, head injury, or seizures. 

Hypoglycemic hemiplegia is a rare event in the pediatric diabetes literature, and its association with exercise as a trigger is even rarer. The reported clinical presentation in most cases involves right-sided hemiplegia, and this may be flaccid or spastic in nature [1, 2]. Patients may be awakened by these episodes, which suggest sleep-onset-related mechanism [1, 3–5]. Unlike our patient, where intravenous dextrose administration resulted in rapid recovery, it may take up to 24 hours for motor function to return to normal [6, 7]; recurrence of hemiplegia is rare [3]. 

Investigations show these children to have normal brain imaging studies and angiograms [2, 6], but the electroencephalogram may show slow-wave activity [6]. The prognosis is excellent, with no residual neurological or cognitive deficits [2, 3, 7]. With this in mind, and in the absence of a precipitating cause, we withheld further investigations. 

In insulin-treated diabetes, exercise-induced hypoglycemia results from a mismatch between carbohydrate intake, exercise patterns, and insulin requirements. Hypoglycemia may develop when carbohydrate intake is less than what is needed for a particular physical activity; this may occur when the child does not consume the recommended meals or snacks before or during exercise. Importantly, the inadequate repletion of glycogen stores after exercise is a common preventable cause of hypoglycemia. 

Insulin is already given when children start to exercise; as exercise continues, there will be relative excess of insulin to carbohydrate load if no insulin dose adjustment is made and no carbohydrates are ingested, resulting in hypoglycemia [8]. Unpredictable insulin absorption with activity may also contribute to hypoglycemia; as the skin temperature rises with increased vascular supply, insulin absorption and action may be enhanced [9]. 

Exercise increases muscle insulin sensitivity [10, 11]; it also enhances non-insulin-dependent glucose shunting into muscle as a source of fuel for exercising muscle [12]. In addition, increased consumption of carbohydrates by muscle with activities of unusual type, intensity, and duration increases the risk of hypoglycemia. 

Clinically, hypoglycemia presents with cardiovascular, neuropsychological, and cognitive manifestations; if unrecognized and untreated, it can progress to coma and death. The presentation of prepubertal diabetic children is characterized by more neuroglycopenic than autonomic symptoms; the former may include confusion, irritability, aggression, argumentation, and moodiness [5, 13]. In our patient, the presentation was different with neuroglycopenia featuring as hemiplegia, with no other neuroglycopenic or autonomic features noted. 

The mechanism of hypoglycemic hemiplegia is not clear, but effects of hypoglycemia on motor neurons are hypothesized to be an important factor. While both exercise and excess insulin may lead to hypoglycemia, it is unclear if the mechanism leading to hypoglycemia impacts cortical motor neurons differently. 

Recent evidence has shown equal glucose uptake and metabolism by cerebral neurons in hypoglycemia, and this suggests that impaired motor neuronal function may be mediated via mechanisms that are not solely dependent on glucose supply, but may be related to impaired signaling pathways or intracellular molecule level or function with hypoglycemia [14, 15]. 

As mentioned earlier, hypoglycemia triggers neuroendocrine and autonomic responses. In diabetic children these responses are more vigorous than in diabetic adults, but the neuroendocrine response to hypoglycemia is impaired, with reduced catecholamine, growth hormone, and glucagon surges [16]. Excellent glycemic control usually leads to intermittent hypoglycemia that, even if mild or transient, may further impair the neuroendocrine counterregulatory response, resulting in more severe hypoglycemia [17, 18]. 

In addition, both sympathetic and parasympathetic arms of the autonomic nervous system are involved in response to hypoglycemia and are accelerated by neuroendocrine response with production of adrenaline. Autonomic responses may also be impaired, and coupled with suboptimal neuroendocrine responses, may contribute to hypoglycemia [13].

In summary, we report a 10-year-old boy with exercise-induced hypoglycemic hemiplegia. Complete recovery of neurological function was noted with intravenous dextrose infusion. Physical activity is highly encouraged in diabetic children, but it is important that they are safe while exercising. 

Frequent blood glucose monitoring and adherence to regular meals and snacks before, during, and after activities are essential [8]. Families and supervising adults need to be educated about the effects of exercise on blood glucose and have plans to anticipate, prevent, and treat hypoglycemia. While hypoglycemic hemiplegia is rare, it is a frightening experience to caregivers, and efforts should concentrate on its prevention by preventing hypoglycemia. 
